# Autologous Stem Cells Combined Core Decompression for Treatment of Avascular Necrosis of the Femoral Head: A Systematic Meta-Analysis

**DOI:** 10.1155/2017/6136205

**Published:** 2017-08-03

**Authors:** Shibing Xu, Lei Zhang, Hongting Jin, Letian Shan, Li Zhou, Luwei Xiao, Peijian Tong

**Affiliations:** ^1^The First Clinical Medical College of Zhejiang Chinese Medical University, Hangzhou, Zhejiang 310053, China; ^2^Zhejiang Chinese Medical University, Hangzhou, Zhejiang 310053, China; ^3^Institute of Orthopaedics and Traumatology of Zhejiang Province, Hangzhou, Zhejiang 310053, China; ^4^Department of Orthopaedic Surgery, The First Affiliated Hospital of Zhejiang Chinese Medical University, Hangzhou, Zhejiang 310006, China

## Abstract

**Objective:**

This study aims to systematically evaluate the efficacy and safety of core decompression combined transplantation of autologous bone marrow stem cells (CDBMSCs) for treatment of avascular necrosis of the femoral head (ANFH).

**Methods:**

Randomized controlled trials (RCTs) regarding effectiveness of core decompression combined transplantation of autologous bone marrow stem cells for treating ANFH were searched in 8 comprehensive databases prior to September 2016. The data analysis was performed by using the RevMan version 5.3.

**Results:**

A total of 11 studies with 507 participants were included. Results showed that CDBMSCs group was more effective than CD group in increasing Harris hip score, decreasing necrotic area of femoral head, collapse of femoral head, and conversion to total hip replacement incidence. In the subgroup analysis, the results did not change in different intervention measure substantially. In addition, the safety of CDBMSCs for ANFH is reliable.

**Conclusion:**

Based on the systematic review, our findings suggest that core decompression combined transplantation of autologous bone marrow stem cells appeared to be more efficacious in the treatment at early stages of ANFH.

## 1. Introduction

Avascular necrosis of the femoral head (ANFH) is a progressive pathological process, usually caused by disruption of the blood supply to the femoral head and elevation of intraosseous pressure. Although the pathogenesis of ANFH remains uncertain [1, 2], it is generally received that the variety of etiologies determined the already precarious circulation of the femoral head, resulting in bone ischemia triggering the death of bone cells and eventually collapse of the necrotic segment [3–5]. The disease usually progresses to femoral head collapse and secondary symptomatic hip arthritis [6, 7]. In most patients without effective early treatment, this type of osteonecrosis can develop into femoral head collapse with subsequent hip joint destruction and eventually require total hip arthroplasty (THA) to restore joint function [8]. This condition usually affects the young patients. However, the THA cannot be expected to increase the patient's lifetime, so the hip-preserving treatments are especially important for these patients in early stage of ANFH [9, 10]. There is a number of treatment options that have been used to prevent or delay the progress of the diseases towards femoral head collapse. Core decompression (CD) is a commonly used method for treating the early stages of ANFH. It is believed that core decompression leads to a reduction in the intraosseous pressure and also stimulates stem cell regeneration. But the outcome of CD is variable and is still controversial [11–13]. This is attributed to the relative insufficiency of osteoprogenitor cells in the proximal femur of the osteonecrotic hip [14, 15]. Recent research has focused on explaining the molecular mechanisms involved in the pathogenesis of ANFH [16–18]. Bone marrow stem cells (BMSCs) have the ability to maintain multiple cell lineages while being capable of differentiating into various cellular types, such as osteocytes, chondrocytes, osteoblasts, and adipocytes [19–21]. It had been shown that bone marrow stem cells implantation into the necrotic lesion of the femoral head is a promising cellular-based therapy [22, 23]. Various studies report the clinical application injection of the BMSCs into the CD hole in patients having improved clinical success in the treatment of precollapse hips [24–28]. This treatment concept was CD reduction in the intraosseous pressure and BMSCs could be reinjected to the trabeculae of the necrotic zone within the femoral head, enhancing regeneration and remodeling of the necrotic bone [29].

We decided to conduct the latest systematic review to investigate whether implantation of autologous bone marrow stem cells into the core decompression track would improve the clinical efficacy of ANFH compared to the classical method of core decompression alone. The outcomes of interest were Harris hip score, necrotic area of femoral head, collapse of femoral head, and conversion to total hip replacement.

## 2. Materials and Methods

### 2.1. Search Strategy

The following electronic databases were searched from their available dates of inception to the latest issue, September 2016: PubMed, Embase, Web of Science, Cochrane Library, China Knowledge Resource Integrated Database (CNKI), VIP Database, Wan fang Database, and Chinese Biomedical (CBM) Literature Database. The search terms were used as follows: (1) Femur Head Necrosis or Avascular Necrosis of Femur Head or Aseptic Necrosis of Femur Head and (2) core decompression or center decompression and (3) Bone marrow mesenchymal stem cells or bone marrow stem cell or bone marrow mononuclear cell. To collect enough tests, related publications' list of references is determined by searching for additional research. There were no language restrictions on trial eligibility.

### 2.2. Selection Criteria

Studies were included if they met the following criteria: (1) study design was an RCT; (2) patients diagnosed with ANFH according to the Association of Research Circulation Osseous (ARCO) classification or other criteria was deemed reasonable; and (3) the intervention was experimental groups receiving CD combined with BMSCs for the treatment of ANFH and control groups with CD treatment. In addition, the experiment group which received CDBMSCs combined with the certain therapy (e.g., autologous bone grafting) and the control group with the same certain therapy were both included.

Studies were excluded if they met the following criteria: (1) randomized crossover trials, case reports, reviews, qualitative studies, or animal experiments; (2) the studies which did not contain the effectiveness comparison between experiment group and the control group for treating ANFH; (3) participants receiving treatment which were CD combined with other treatments (e.g., Tantalum rod, vascularized pedicle bone flap) or transplantation of stem cells not derived from bone marrow.

### 2.3. Data Extraction and Analysis

All data were independently abstracted by two investigators (Shibing Xu and Lei Zhang) using a predefined data extraction form. Extracted information included first author's name, publication year, sample sizes, patient characteristics, methodological features of the studies, quality of trial design, interventions, main outcome assessments, follow-up time, and withdrawal. If the required information was not available in the included studies, we contacted the original authors by email. Disagreement was resolved by discussion or consensus with a third reviewer (Peijian Tong).

The risk of bias tool of the Cochrane Collaboration was applied by two independent authors (Shibing Xu and Lei Zhang), including the following domains: sequence generation, allocation sequence concealment, blinding of participants and personnel and outcome assessors, incomplete outcome data, selective outcome reporting, and other sources of bias. The assessments were classified into three levels: low risk, high risk, and unclear risk [41].

Statistical analyses were performed with Review Manager 5.3 software (Cochrane Community, London, United Kingdom). Dichotomous data were presented as odds ratio (OR) and continuous outcomes as mean difference (MD), both with 95% confidence interval (CI). Fixed effects model was applied to analyze data if there was low heterogeneity (*P* > 0.10, *I*^2^ ≤ 50%); random effects model was used if there was high heterogeneity (*P* < 0.10, *I*^2^ ≥ 50%). Data were not pooled if there was significant heterogeneity, in which case we explored potential causes of heterogeneity by conducting subgroup analyses. Sensitivity analysis would be performed by omission of each study to evaluate stability of the results if heterogeneous studies existed. Funnel plots were used to assess publication bias. All tests were two-tailed and *P* value < 0.05 was deemed statistically significant [42].

We used the Grades of Recommendations Assessment, Development, and Evaluation (GRADE) to assess the level of evidence and summarize each outcome. The following domains were assessed: risk of bias, consistency, directness, precision, publication bias, and additional points. The assessments were classified into four levels: high, moderate, low, or very low. Summary tables were constructed using the GRADE Profiler (version 3.6) [43, 44].

## 3. Results

### 3.1. Study Selection and Study Characteristics

**Figure 1:**
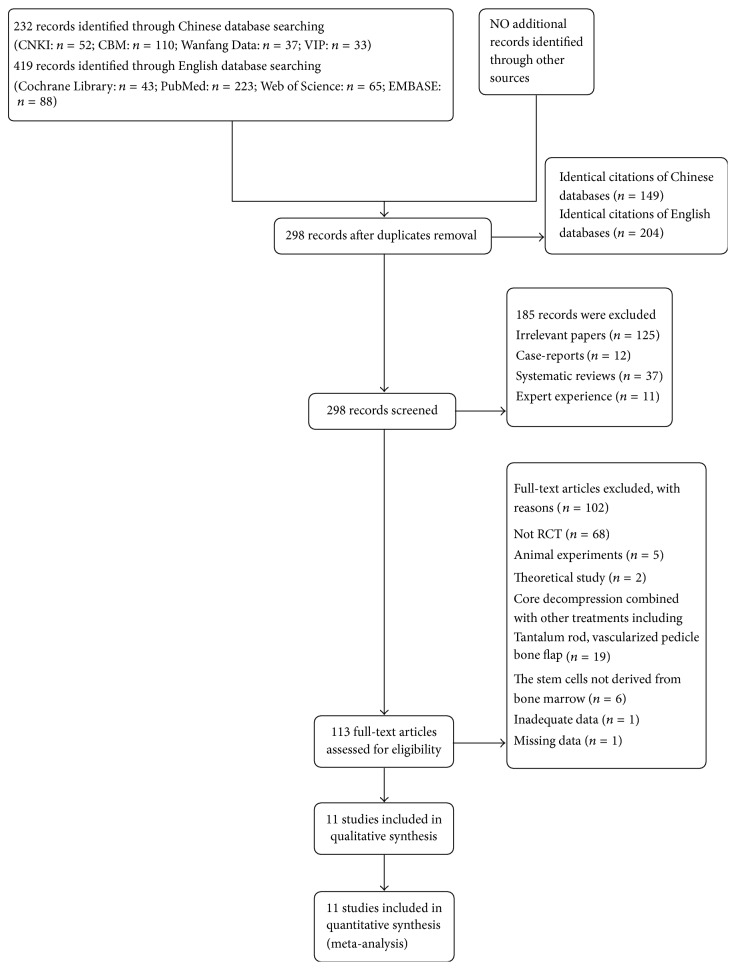
Flow diagram of the study selection procedure.

We identified 651 references (232 records from Chinese databases and 419 records from English databases) through electronic searches. In total, 11 RCTs with 507 enrolled participants were included (Figure 1). The characteristics of the included trials are summarized in Table 1. Five studies were published in Chinese and six studies in English between 2008 and 2016.

Participants were diagnosed with ANFH stage via three different criteria: the ARCO diagnostic criteria were used in nine studies [30–34, 36, 38–40], the Ficat diagnostic criteria were used in one study [35], and the Steinberg diagnostic criteria were used in one study [37]. In each of the included studies, baseline difference between experiment group and control group revealed no statistical significance. The interventions for the experiment groups included CD combined with BMSCs therapy [30–34, 36, 38–40] and CD combined with BMSCs plus autologous bone grafting [35, 37]. In the control groups, CD treatment was used in eight studies [31–34, 36, 38–40], CD combined with autologous bone grafting was adopted in two studies [35, 37], and CD combined with unprocessed bone marrow was used in one study [30]. Harris hip score was reported in six studies [32, 36–40]. Necrotic area of femoral head was reported in four trials [36–38, 40]. Nine studies [30, 31, 33–35, 37–40] reported the outcomes of collapse of the femoral head. Seven studies [30, 31, 33–35, 39, 40] provided the number of hips in patients with conversion to THR. As for adverse events, seven studies [32–36, 39, 40] reported that no complication was observed in the patients during or after the treatment; four studies [30, 31, 37, 38] did not mention the adverse events.

### 3.2. Risk of Bias Assessment

**Figure 2:**
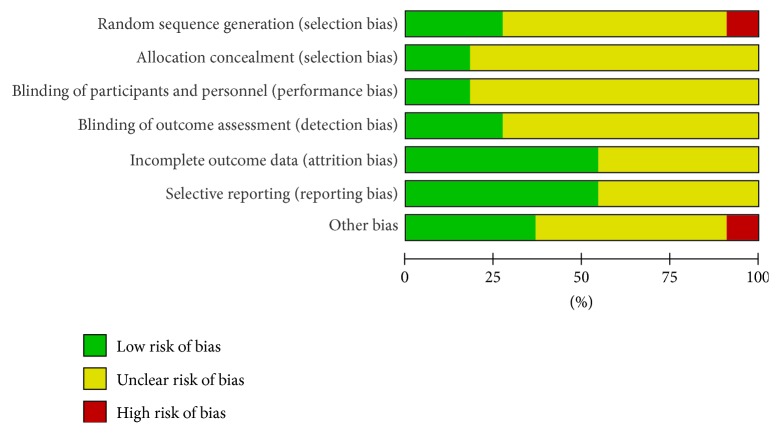
Quality assessment of included studies. Risk of bias graph.

The risk of bias assessment is depicted in Figure 2. The methodological quality of the trials varied stably. Most of them possibly suffered from selection bias (due to lack of random generation and concealment of the allocation sequence); although all these trials reported randomization, only four adequately described the randomization method: one with a random number table [36] and two using random sequences generation [34, 35] and one study [31] of high risk according to the sequence of seeing the doctor. Only one trial [33] mentioned used sealed envelope for allocation concealment and the remaining trials did not report it. Most of them possibly suffered from performance bias and detection bias (due to poor blinding of participants, personnel, or outcome assessors); two trials [33, 35] mentioned that they had double-blinded participants or personnel. Three trials [33–35] mentioned that they were blinded to outcome assessment. Two studies [32, 34] reported the patient losses to follow-up because the patients did not report the scheduled date and family relocation. We found no other biases in these trials. All reports mentioned that the research was approved by ethics committee and that there was informed consent of patients.

### 3.3. Effect of the Interventions

#### 3.3.1. Harris Hip Score

Six trials [32, 36–40] assessed Harris hip score. According to the different intervention measure, we divided them to two subgroups. In the five studies [32, 36, 38–40] of the CDBMSCs versus CD subgroup, the data was analyzed using a fixed effects model based on the moderate heterogeneity test result (*P* = 0.13, *I*^2^ = 44%). After combining the data and analyzing them, the result showed that CDBMSCs were more effective than CD alone in increasing Harris hip score (MD = 11.28, 95% CI = 9.52 to 13.03, *P* < 0.00001). While in the CDBMSCs plus ABG versus CD plus ABG subgroup [37], the result showed that experiment group was more effective than control group (MD = 12.11, 95% CI = 9.41 to 14.81, *P* < 0.00001).

**Figure 3:**
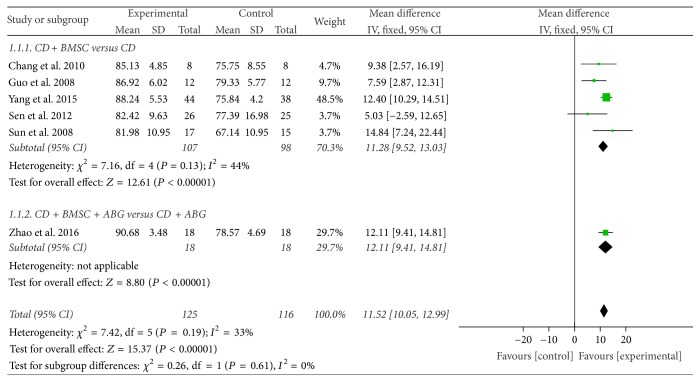
Forest plot of experimental treatment versus control group interventions on Harris hip scores according to intervention measure.

After the test of heterogeneity between the subgroups, no heterogeneity test result between them (*P* = 0.61, *I*^2^ = 0%) was found. Accordingly, the results of the two subgroups could be merged. The combined results indicated that CDBMSCs therapy was significantly superior to CD treatment in increasing Harris hip score of the femoral head (MD = 11.52, 95% CI = 10.05 to 12.99, *P* < 0.00001) (Figure 3).

#### 3.3.2. Necrotic Area of Femoral Head

Necrotic area of femoral head was available in four trials [36–38, 40]. We divided them to two subgroups according to the different intervention measure. In the three studies [36, 38, 40] of the CDBMSCs versus CD subgroup, meta-analysis revealed that CDBMSCs were more effective than CD in decreasing necrotic area of the femoral head (MD = −5.52, 95% CI = −7.07 to −3.97, *P* < 0.00001). The result was homogenous (*P* = 0.64, *I*^2^ = 0%) and a fixed effects model was applied, while in the CDBMSCs plus ABG versus CD plus ABG subgroup [37], the result showed that experiment group was more effective than control group (MD = −7.37, 95% CI = −13.03 to −1.71, *P* = 0.01).

**Figure 4:**
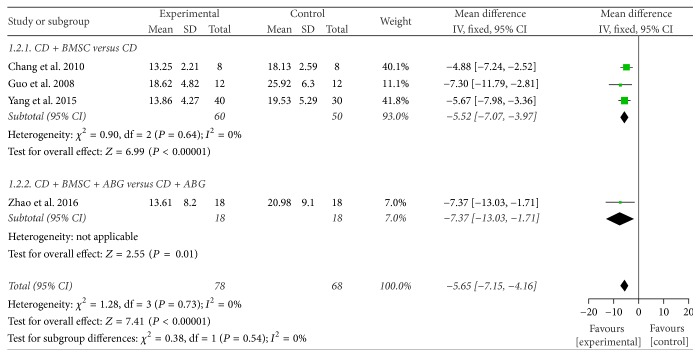
Forest plot of experimental treatment versus control group interventions on postoperation necrotic area according to intervention measure.

After the test of heterogeneity between the subgroups, no significant difference between them (*P* = 0.54, *I*^2^ = 0%) was found. So, the results of the two subgroups could be merged. The combined results showed that CDBMSCs therapy was significantly superior to CD treatment in decreasing necrotic area of the femoral head (MD = −5.65, 95% CI = −7.15 to −4.16, *P* < 0.00001) (Figure 4).

#### 3.3.3. Collapse of the Femoral Head

Nine trials [30, 31, 33–35, 37–40] mentioned the numbers of collapse instances of the femoral head through follow-up. In the 7 studies [30, 31, 33, 34, 38–40] of the CDBMSCs versus CD subgroup, the result showed that CDBMSCs were more effective than CD in decreasing collapse of the femoral head, with a statistically significant difference between the two groups (OR = 0.22, 95% CI = 0.10 to 0.50, *P* = 0.0003). As there was no homogeneity in the consistency of the trial results (*P* = 0.78, *I*^2^ = 0%), a fixed effects model was applied, while in the CDBMSCs plus ABG versus CD plus ABG subgroup, the result showed that experiment group almost reached borderline levels of statistical significance compared to the control group (OR = 0.27, 95% CI = 0.07 to 1.09, *P* = 0.07). The heterogeneity between the two studies [35, 37] had no significant difference (*P* = 0.94, *I*^2^ = 0%); a fixed effects mode could be used.

**Figure 5:**
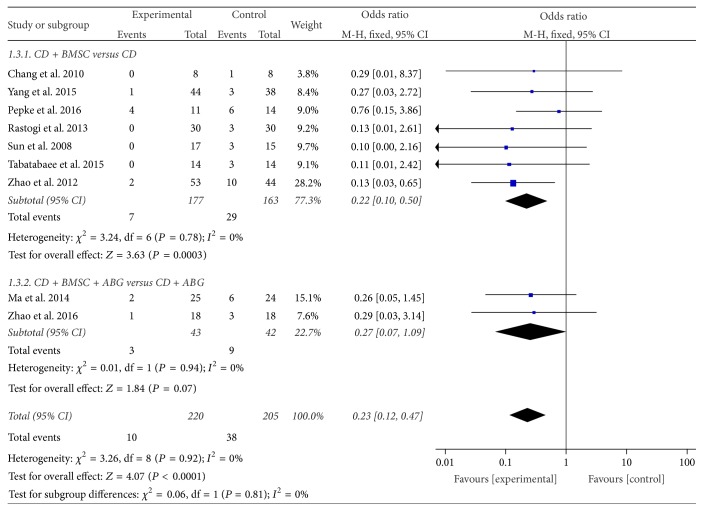
Forest plot of experimental treatment versus control group interventions on collapse of femoral head.

After the test of heterogeneity between the subgroups, no significant difference between them (*P* = 0.81, *I*^2^ = 0%) was found. Accordingly, the results of the two subgroups could be merged. The combined results indicated that CDBMSCs therapy was significantly superior to CD treatment in decreasing collapse of the femoral head (OR = 0.23, 95% CI = 0.12 to 0.47, *P* < 0.0001) (Figure 5).

#### 3.3.4. Conversion to THR

**Figure 6:**
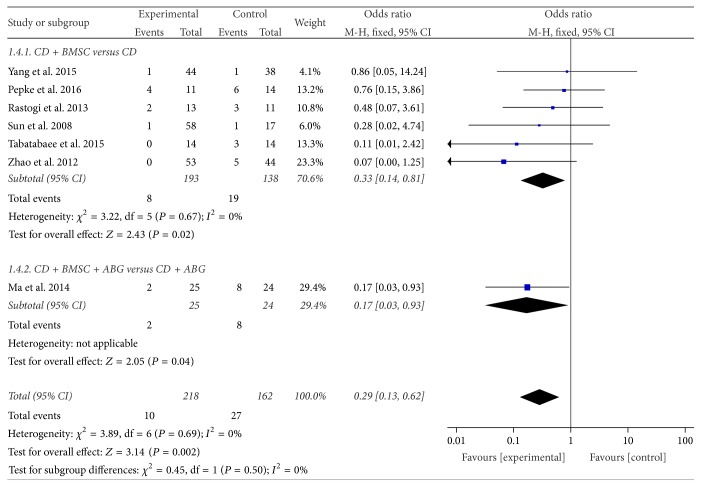
Forest plot of experimental treatment versus control group interventions on THA of femoral head.

Seven studies [30, 31, 33–35, 39, 40] evaluated situation of conversion to THR at the period of follow-up. In the 6 studies [30, 31, 33, 34, 39, 40] of the CDBMSCs versus CD subgroup, the pooled results showed a significant decrease in conversion to THR in the CDBMSCs group compared with the control groups (OR = 0.33, 95% CI = 0.14 to 0.81, *P* = 0.02). Owing to no heterogeneity (*P* = 0.67, *I*^2^ = 0%), a fixed effects model could be applied, while in the CDBMSCs plus ABG versus CD plus ABG subgroup [33], the result was similar (OR = 0.17, 95% CI = 0.03 to 0.93, *P* = 0.04). After the test of heterogeneity between the subgroups, no significant difference between them (*P* = 0.50, *I*^2^ = 0%) was found. Accordingly, the results of the two subgroups could be merged. The combined results showed that CDBMSCs therapy was significantly superior to CD treatment in the conversion to THR incidence of the femoral head (OR = 0.29, 95% CI = 0.13 to 0.62, *P* = 0.002) (Figure 6).

### 3.4. Adverse Events

Seven [32–36, 39, 40] of the eleven studies reported no adverse effects after operation in experiment group and control group. The remaining four studies [30, 31, 37, 38] did not mention whether or not there were adverse reactions. In a word, safety of CDBMSCs for ANFH is acceptable.

### 3.5. Publication Bias Analysis

Funnel plot was used to check for the existence of publication bias, because the sample sizes of this meta-analysis were too small to detect publication bias.

### 3.6. Level of Evidence

The levels of evidence as determined by GRADE were low (Table 2). Most of the studies did not report blinding, randomization sequence generation, or allocation concealment methods, so all outcomes were initially downgraded. In addition, the small number of participants of all outcomes also downgraded all outcomes except collapse of the femoral head.

## 4. Discussion

Core decompression (CD) is an easy-performed and popular procedure which has been used for the treatment of osteonecrosis for approximately three decades [45, 46]. It is generally believed that core decompression works by reducing elevated intraosseous pressure and restoring vascularity of the femoral head, therefore preventing neurovascular compression and promoting healthy new bone formation [47]. However, the results of core decompression alone usually deteriorate with more advanced lesions. So we need to take further reconstructive intervention. Recent research has focused on the role of BMSCs in the pathogenesis of osteonecrosis. Such cells were found to be decreased in number and activity in osteonecrotic femoral heads. These findings promoted researchers to develop a new approach for the treatment of ANFH, based on combination with core decompression implantation BMSCs into the necrotic zone of the femoral head. BMSCs show multipotential capacities to differentiate into osteoblasts, hemangioblasts, and endothelial cell progenitors, which function to repair the necrosis region of the femoral head [48, 49]. In addition, researchers [50, 51] have shown that BMSCs also release a variety of growth factors to facilitate bone regeneration. They also enhance vascularization and oxygen flow to the ischemic tissues and accelerate fracture healing [52]. In a study by Song et al. [53], histologic evidence of new bone formation in the femoral head after 6 weeks of mesenchymal cell transplantation has been shown. Instillation of bone marrow stem cells along with CD in osteonecrosis of the femoral head was pioneered by Hernigou et al. in 2006 [54]. The effectiveness of autologous cell therapy is highly related to the stage of the disease and also to the number of BMSCs transplanted. They showed that when patients were operated upon before collapse of the ANFH ensued and when they received a greater number of BMSCs in the autologous bone marrow injected into the necrotic lesion, a more favorable outcome could be expected.

The previous meta-analyses [55, 56] concluded that there were limited evidence to prove the effectiveness of CDBMSCs for treating ANFH due to having a small sample size and low methodological quality. Therefore, a more clear evaluation on the CD combined BMSCs treatments in ANFH is essential. In the present meta-analysis, we restricted our high-quality RCTs to CDBMSCs therapy for ANFH. In addition, we compared posttreatment indexes of the main outcomes including Harris hip score, necrotic area of femoral head, collapse of the femoral head, and conversion to THR in our analysis, which might contribute to more objective conclusions.

In this systematic review, according to inclusion criteria, 11 studies of CDBMSCs for ANFH were eligible for our systematic review and meta-analysis. This systematic review found that BMSCs implantation into the core decompression track resulted in better clinical outcomes of ANFH than core decompression treatment, as it was found to markedly improve Harris hip score, reduce necrotic area of femoral head, delay the progression of the disease to the stage of femoral head collapse, and decrease the need for total hip arthroplasty. In the subgroup analysis, the results did not change obviously in different intervention methods. Moreover, there was sparse information in these RCTs regarding the processes of randomization and allocation concealment, and only one [34] of the RCTs blinded the statisticians which may have led to a considerable risk of bias. Taking into account the small sample sizes of the included trials, it was difficult to make robust conclusions.

Sources of clinical heterogeneity included sex, age, stage of the disease, etiology of ANFH, and surgical intervention. Some sources of clinical diversity can be addressed by appropriate subgroup analysis. We conduct subgroup analysis to explore the heterogeneity of treatment effects in RCTs according to type of surgical intervention, but, patients characteristics, specific interventions, and follow-up time were not restricted uniformly.

In this review, seven of the eleven studies reported no adverse effects after operation in experiment group and control group, whereas the remaining studies did not mention whether or not there were adverse reactions. Therefore, we have to think roughly that safety of CDBMSCs for ANFH is acceptable. Future clinical trials containing a larger simple size and long follow-up time are required to evaluate safety of CDBMSCs for ANFH.

There are some limitations that should be taken into consideration when accepting the findings of this review. Firstly, the vast majority of the included trials failed to describe detailed information about randomization, allocation concealment, and blinding, as these are the core standards of a well-designed RCT [57, 58]. It is so hard to randomly allocate the patients' hip joint that most of clinical studies failed to randomize. These reasons were contributed to bias of risk of included studies. Secondly, all trials reported positive effects in the CDBMSCs for the treatment of ANFH, while negative findings are less likely to be published, implying that publication bias may have existed. Thirdly, except for one study [35], the remainder of the studies ignored the sample size estimation. All included studies were of small sample sizes, which weakened the validity of statistical analysis. Therefore we should be cautious about the results of the meta-analysis. Last but not least, we failed to generate a funnel plot for outcomes to detect potential publication bias due to the limited number of included trials.

## 5. Conclusion

This systematic review found that BMSCs implantation into the core decompression track appeared to be more efficacious in the treatment of ANFH than core decompression only, delayed ANFH progression, reduced necrotic area of femoral head, decreased the need for total hip arthroplasty, and improved Harris hip score. However, more rigorously designed and higher quality trials with larger sample size are necessary for better confirming the effectiveness of CD combined with BMSCs on ANFH.

## Figures and Tables

**Table 1:** Characteristics of the included studies in the meta-analysis.

Study	Sample size (hips)	Age (years)	Diagnostic criteria	Disease stages (hips)	Etiological factors (hips)	Intervention	Outcome	Follow-up (months) Median
Rastogi et al., 2013 [30]	E: NA (30)	E: 34.7 ± 7	ARCO	I/II/III	E: S (10), A (2), I (14), Sm (4)	E: CD + BMSCs	CFH, CT	24
	C: NA (30)	C: 33 ± 7.7			C: S (8), A (6), I (12), Sm (4)	C: CD + UBM		
Pepke et al., 2016 [31]	E: NA (11)	E: 44.3 ± 3.4	ARCO	II	NA	E: CD + BMSCs	CFH, CT	24
	C: NA (14)	C: 44.5 ± 3.3				C: CD		
Sen et al., 2012 [32]	E: NA (26)	NA	ARCO	I/II (NA)	Tr (17), S (20), A (8)	E: CD + BMSCs	Harris	24
	C: NA (25)				I (2), P (2), Cs (2)	C: CD		
Tabatabaee et al., 2015 [33]	E: 14 (14)	E: 37 ± 11.4	ARCO	E: I (3), II (9), III (2)	E: S (10), I (4)	E: CD + BMSCs	CFH, CT	24
	C: 13 (14)	C: 26.8 ± 5.8		C: I (2), II (7), III (5)	C: S (9), I (5)	C: CD		
Zhao et al., 2012 [34]	E: 50 (53)	E: 32.7 ± 10.5	ARCO	E: IC (3), IIA (15), IIB (24), IIC (11)	E: Tr (8), S (11), A (11), Cd (6), I (17)	E: CD + BMSCs	CFH, CT	60
	C: 50 (51)	C: 33.8 ± 7.7		C: IC (2), IIA (15), IIB (22), IIC (12)	C: Tr (12), S (13), A (8), Cd (5), I (13)	C: CD		
Ma et al., 2014 [35]	E: 21 (25)	E: 35.6 ± 8.1	Ficat	E: I (3), II (17), III (5)	E: S (15), A (4), I (6)	E: CD + ABG + BMSCs	CFH, CT	24
	C: 18 (24)	C: 34.8 ± 11.5		C: I (4), II (15), III (5)	C: S (15), A (3), I (6)	C: CD + ABG		
Guo et al., 2008 [36]	E: 10 (12)	42 (28~61)	ARCO	III (24)	S (7), A (4), Tr (3), U (6)	E: CD + BMSCs	Harris, PMN	12
	C: 10 (12)					C: CD		
Zhao et al., 2016 [37]	E: 18 (NA)	20~42 (30.5)	Steinberg	NA	NA	E: CD + BMSCs + ABG	Harris, PMN, CFH	24
	C: 18 (NA)					C: CD + ABG		
Yang et al., 2015 [38]	E: 30 (44)	E: 16~45 (35.3)	ARCO	E: I (16), II (28)	Tr (6), S (36), A (38), U (2)	E: CD + BMSCs	Harris, PMN, CFH, CT	18
	C: 26 (38)	C: 18~46 (37.6)		C: I (16), II (22)		C: CD		
Sun et al., 2008 [39]	E: 15 (17)	17~47 (36)	ARCO	I (8), II (24)	Tr (7), S (9), A (10), U (2)	E: CD + BMSCs	Harris, CFH, CT	18
	C: 13 (15)					C: CD		
Chang et al. 2010 [40]	E: 8 (8)	19~43 (35.7)	ARCO	E: IIA (2), IIB (2), IIC (3), IIIA (1)	S (2) A (3) U (3)	E: CD + BMSCs	Harris, PMN, CFH	12
	C: 8 (8)			C: IIA (4), IIB (2), IIC (1), IIIA (1)		C: CD		

*Note*. E: experiment group. C: control group. S: steroids. A: alcohol abuse. Tr: trauma. I: idiopathic. P: pregnancy. Cs: Cushing. Cd: caisson disease. Sm: smoking. U: unknown aetiology. ARCO: Association Research Circulation Osseous. CD: core decompression. BMSCs: bone marrow stem cells. UBM: unprocessed bone marrow. ABG: autologous bone grafting. PMN: postoperation MRI necrosis area. CFH: collapse of the femoral head. CT: conversion to THR. NA: not available.

**Table 2:** Level of evidence (GRADE).

Outcome	Effect		Number of participants	Quality of the evidence
	Relative effect (95% CI)	Absolute effect (95% CI)	(studies)	(GRADE)
Harris hip score		MD 11.52 higher (10.05 to 12.99 higher)	241 (6 studies)	⊕⊕OO Low^1,2^

Necrotic area of femoral head		MD 5.65 higher (7.15 to 4.16 higher)	146 (4 studies)	⊕⊕OO Low^1,2^

Collapse of the femoral head	OR 0.23 (0.12 to 0.47)	136 fewer per 1000 (from 89 more to 1599 more)	425 (9 studies)	⊕⊕OO Low^1,2^

Conversion to THR	OR 0.29 (0.13 to 0.62)	112 fewer per 1000 (from 56 more to 141 more)	380 (7 studies)	⊕⊕OO Low^1,2^

^1^ Most of them did not mention randomization process, allocation concealment, and blinding; ^2^ published evidence is limited due to a small number of trials, all of which are showing benefits.
